# Age-Related changes in the morphological features of medial column of the proximal humerus in the Chinese population

**DOI:** 10.3389/fsurg.2023.1138620

**Published:** 2023-03-03

**Authors:** Zuhao Chang, Zhengguo Zhu, Wei Zhang, Hua Chen, Yujie Liu, Peifu Tang

**Affiliations:** ^1^ Department of Orthopedics, Chinese PLA General Hospital, Beijing, China; ^2^ AI Sports Engineering Lab, School of Sports Engineering, Beijing Sport University, Beijing, China

**Keywords:** medial column, proximal humerus, morphological features, age-related changes, morphological grading, computed tomography

## Abstract

**Background:**

Age-related changes in the medial column (MC) of the proximal humerus have a major impact on fracture management; however, the changes in the morphological features remain unclear. This study aimed to investigate the age-related changes in the morphological features of MC and present the morphological grading.

**Methods:**

One hundred computed tomography (CT) images of the proximal humerus of 100 individuals (19–95 years) were retrospectively obtained. The individuals were categorized into five age groups to quantify the differences among different ages; the youngest group (18–44 years) served as the baseline group. Parameters of the morphological features were measured on CT images with multiplanar reconstruction based on an explicit definition of MC, including length, thickness, width, oblique thickness (D_SM_), humeral head diameter (D_HM_), and ratio (R_SM_) of D_SM_ to D_HM_. The morphological grading of MC was presented based on the value of R_SM_ deviating different standard deviations (SD) from the mean value in the baseline group.

**Results:**

Significant negative correlations were observed between age and the morphological parameters of MC (r ranged from −0.875 to −0.926; all *P* < 0.05), excluding D_HM_ (*r* = 0.081, *P* = 0.422). Significant differences in the values of morphological feature parameters were detected among the five age groups (all *P* < 0.001). The highest mean values of morphological feature parameters were observed in the youngest group (18–44 years), which decreased gradually with increasing age until the lowest mean values were observed in the oldest group (≥90 years) (all *P* < 0.05). The morphological features of MC were categorized into three grades based on the value of R_SM_ deviating 1.5 SD or 3 SD from the mean value in the baseline group.

**Conclusion:**

Our study shows that the parameter values of morphological features of MC decreased with increasing age. The morphological features of MC could be categorized into three grades. Our findings may provide a more comprehensive insight into age-related changes in the morphological features of MC that facilitate risk stratification and optimize the management of proximal humeral fractures.

## 1. Introduction

The medial column (MC) is considered the osseous region of the posteromedial metaphysis of the proximal humerus (PH), which plays a crucial role in fracture management (1–3). MC reportedly develops remarkable changes with increasing age owing to the development of osteopenia or osteoporosis (4, 5). These age-related changes in MC may be responsible for the increased risk of proximal humeral fractures (PHFs) and higher fracture severity (1, 4, 6, 7). Furthermore, incompetent MC, i.e., fragile with impaired strength, due to advanced age is also associated with a higher risk of fracture complications (8–12). Conversely, elderly patients with severe osteopenia treated with integrity restoration of MC could achieve a satisfactory prognosis, similar to that of younger patients (13–16). Therefore, investigating the morphological features of MC and elucidating its age-related changes may be conducive to the management of PHFs. However, the specific definition of MC is inconsistent, and age-related changes in its morphological features remain unclear owing to a lack of studies.

The range of region of interest of MC varied in different studies, ranging between 20 and 40 mm below the humeral head based on subjective interpretations (4, 5, 17, 18). The consequent conclusions may be limited given the subjective selection methods. Furthermore, microstructural assessments of the cortical or trabecular bone in the cadaveric bone have been used to analyze the age-related changes in MC; however, these might be insufficient to derive a comprehensive conclusion given the limited sample sizes and differences between ex and *in vivo* samples (4, 5, 19, 20). A study on the *in vivo* imaging of postmenopausal females reported the regional differences in the cortical bone of MC; however, studies on individuals of other ages remain lacking (18). Moreover, no morphological grading of MC based on its age-related changes—such as the Singh index used for the proximal femur—has been proposed (21–23). Therefore, this study aimed to investigate the age-related changes in the morphological features of MC using *in vivo* imaging based on an objective definition of MC and individuals with a broad age range.

## 2. Materials and methods

### 2.1. Study design

This is a cross-sectional study approved by the Ethics Committee of Chinese PLA General Hospital (No. S2021–021–01) and registered in Chinese Clinical Trial Register (ChiCTR2200059524). We retrospectively reviewed 681 CT images of PH of 628 adult individuals in the Picture Archiving and Communication Systems of our institution between December 2019 and December 2021. The demographic and clinical information was obtained from electronic medical records, and all personal records were anonymized prior to data analysis. Owing to the retrospective and anonymous nature of data collection, the requirement for informed consent was waived.

The CT images were obtained with a 256-slice multidetector CT scanner (Philips Healthcare, Amsterdam, Netherlands) and standardized protocol with high-resolution algorithm (120 KV, 0.675 mm slice thickness, 0.335 mm interlayer spacing, 0.8 mm reconstruction slice thickness, 1.0 mm reconstruction interlayer spacing, and 512 × 512-pixel matrix). For the acquisition of *in vivo* CT images of normal intact PHs, the CT images from individuals older than 18 years of age were included. Axial scanning ranged from more than 3 cm superior to the acromion to more than 3 cm inferior to the deltoid tuberosity. The exclusion criteria were as follows: (1) Prior fracture or surgery in PH; (2) bone diseases and skeletal abnormalities, including osteoarthritis, rheumatism, dysplasia, and deformity; (3) diagnosis of metabolic diseases or receiving treatment that could affect bone metabolism, such as Paget's disease and primary hyperparathyroidism; (4) diagnosis of cancer or other malignant diseases; and (5) history of smoking. Thus, 261 CT images of normal PHs from 261 individuals were obtained.

**Figure 1:**
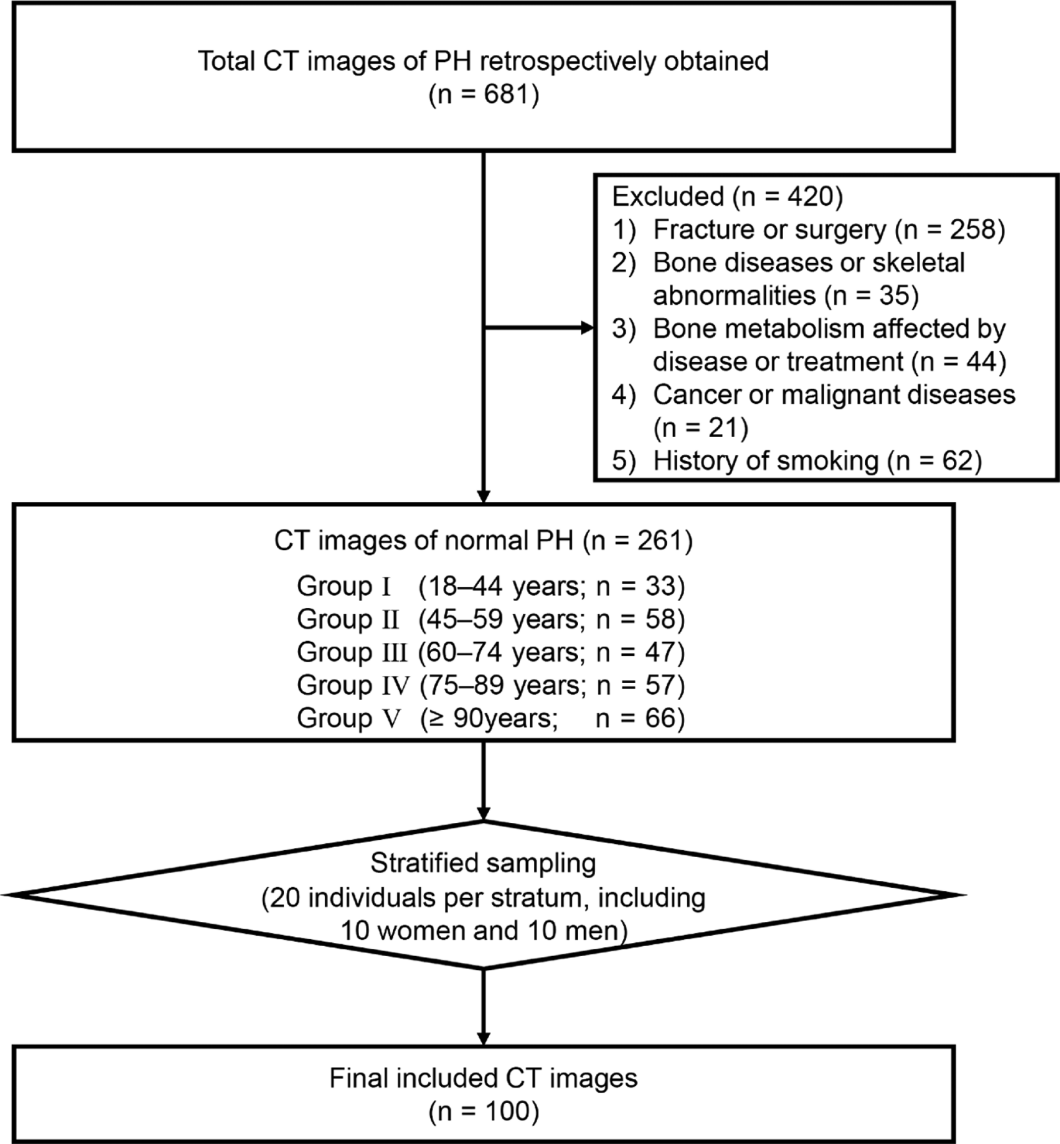
Flow chart of inclusion of computed tomography images of the proximal humerus. CT, computed tomography; PH: proximal humerus.

To further clarify and quantify the age-related changes in the morphological features of MC, age was categorized into five groups (24–26): Group I (18–44 years), Group II (45–59 years), Group III (60–74 years), Group IV (75–89 years), and Group V (≥90 years). Group I served as the baseline group. As the age distribution of individuals was skewed, stratified sampling was used to ensure equal sample sizes among the age groups to avoid the influence of different sample sizes among age strata on further morphological analysis. In total, 100 CT images from 100 individuals were included for analysis (20 individuals per stratum, including 10 women and 10 men; Figure 1). The mean age of individuals included in the study was 64.94 ± 21.22 years (ranges 19–95 years; 64.74 ± 21.22 years for women, and 65.14 ± 21.52 years for men).

### 2.2. Definitions of parameters for morphological features of the mc

**Figure 2:**
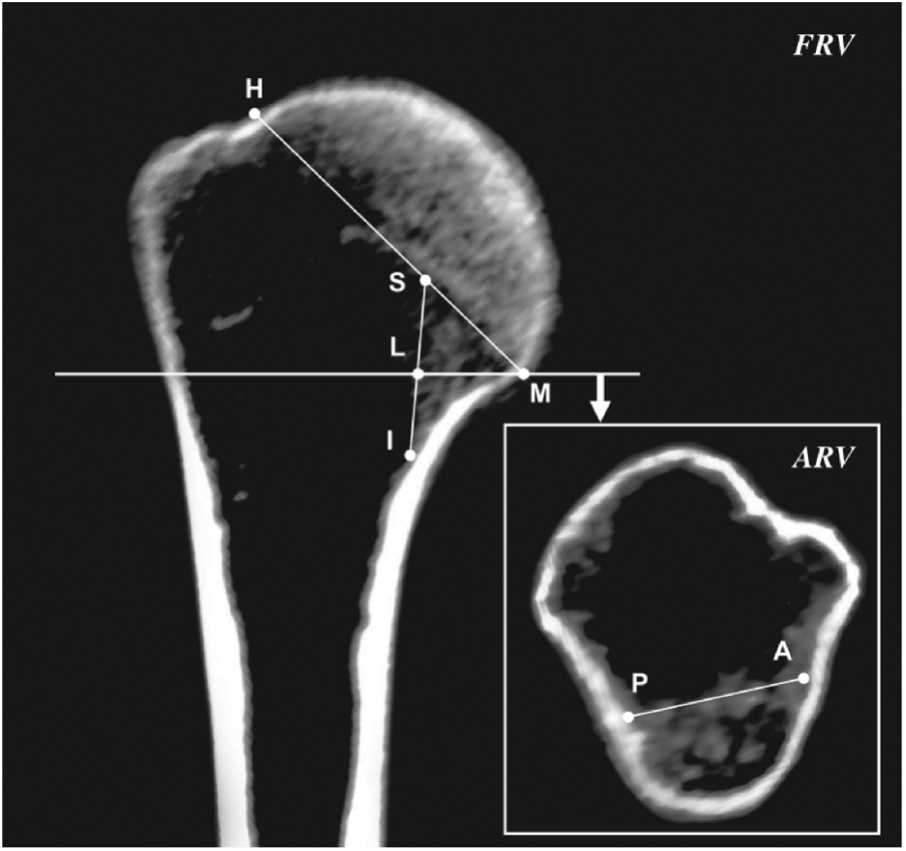
Measurement parameters of the morphological features of the medial column in the frontal and axial reconstruction view. FVR, frontal reconstruction view; ARV, axial reconstruction view.

CT images with multiplanar reconstruction in this study were acquired using RadiAnt DICOM Viewer (version 4.6.5; Medixant, Poznan, Poland). Previous studies showed that the changes in cortical bone and trabecular bone both have an impact on the medial supporting role of MC, and the changes in trabecular bone might be an early sign of a decrease in the mechanical properties of MC (2, 4, 5, 18, 27). Thus, MC was defined as a complex osseous region in the medial metaphysis in this study, comprising the endosteal longitudinal trabecular bone and adjacent cortical bone; the endocortical longitudinal trabecular region was used as a reference (Figure 2). The parameters of the morphological features of MC, defined according to previous studies, were as follows (Figure 2) (7, 28):

(1) The length of MC (D_SI_): The longest axial distance of the endocortical longitudinal trabecular region in the frontal reconstruction view, extending from the intersection of the endosteal surface of the trabecular bone and the epiphyseal line of the humeral head (point S) to the intersection of the endosteal surface of the trabecular bone and distal endocortical surface (point I).
(2) The thickness of MC (D_LM_): The combined horizontal distance of endocortical longitudinal trabecular bone and cortical bone in the frontal reconstruction view, extending from the endosteal surface (point L) to the periosteal surface (point M) at the level of the inferior margin of the humeral head.
(3) The width of MC (D_AP_): The distance between the intersections (point A, point *P*) of the endosteal surface of the endocortical longitudinal trabecular region and antero-posterior endocortical surfaces in the axial reconstruction view of the inferior margin of the humeral head.
(4) The oblique thickness of MC (D_SM_): The sum of the distance between the endocortical longitudinal trabecular region and cortical bone contacting the epiphyseal line in the frontal reconstruction view, extending from point S to point M.
(5) The diameter of the humeral head (D_HM_): The distance between the superior and inferior margins of the humeral head in the frontal reconstruction view, from point H to point M, which indicates the overall size of PH.
(6) The ratio of MC (R_SM_): The ratio of D_SM_ to D_HM_ was calculated to facilitate the comparison of the age-related morphological changes of MC in individuals with different PH sizes.

The measurements of parameters in all individuals were obtained independently by two orthopedic researchers (ZC and WZ) with experience in CT imaging analysis. The intraobserver reliability of the measurement parameters was assessed by repeating the measurements of parameters in all individuals twice randomly by the same researcher (ZC), at least 4 weeks apart. In addition, the measurements of parameters in all individuals were obtained independently by the second researcher (W.Z.) to assess the interobserver reliability of the measurements of parameters. All measurements of parameters in all individuals were obtained thrice, and the average values were taken for final analysis to avoid researcher bias.

The morphological features of MC were graded using R_SM_, as R_SM_ was presented as a ratio that facilitates comparison of the morphological features of MC with different PH sizes. The morphological features of MC were graded based on the value of R_SM_ deviating different standard deviations (SD) from the mean value of R_SM_ in the baseline group (Group I), which was referred to as the morphological grading method of vertebral compressive fractures and the categorization method of bone mineral density (29, 30).

### 2.3. Statistical analysis

The intra- and interobserver reliability of the morphological parameters were assessed using the intraclass correlation coefficient (ICC). The threshold for excellent correlation was set at 0.75 (31). The Shapiro–Wilk test was used as the normality test of continuous variables. The correlation between age and morphological parameters was assessed using Spearman's correlation coefficient. Correlation strength was assessed to be strong at *r* > 0.7, moderate at 0.7 > *r *> 0.3, and weak at *r* > 0.3 (32). The mean values of normally distributed continuous variables within the groups were compared using one-way analysis of variance, followed by the least-significant difference (LSD) post-hoc test for pairwise comparisons. Non-normal distribution data were analyzed using Kruskal–Wallis H test; Bonferroni correction was used in the pairwise comparisons. Categorical variables were presented as constituent ratios and analyzed using the *χ*^2^ test or Fisher's exact test. All statistical analyses were conducted using IBM SPSS Statistics 26.0 (IBM Corp., Armonk, NY, United States), with a *P* value < 0.05 indicating statistical significance.

A priori power analysis (*α* = 0.05, *β* = 0.2, 2-tailed) was performed using PASS (version 15.0.5; NCSS, Kaysville, United States) to achieve a medium to large correlation coefficient (*ρ* ≥ 0.3), which was expected for the correlation between the measured parameters and age. A minimum sample size of 84 individuals was required in the present study.

## 3. Results

### 3.1. Reliability of the measurement parameters of morphological features of MC

The intra- and interobserver reliability showed almost perfect agreement for all measurement parameters of the morphological features in the present study (ICC ranged from 0.814 to 0.952; all *P* < 0.001). Detailed results are presented in Table 1.

**Table 1:** Inter- and intraobserver reliabilities of the measurement parameters.

Parameter	Intraobserver reliability		Interobserver Reliability	
	ICC (95% CI)	*P* value	ICC (95% CI)	*P* value
D_SI_	0.826 (0.786, 0.887)	<0.001	0.834 (0.790, 0.869)	< 0.001
D_LM_	0.905 (0.859, 0.936)	< 0.001	0.910 (0.869, 0.931)	<0.001
D_AP_	0.814 (0.756, 0.882)	<0.001	0.819 (0.777, 0.852)	<0.001
D_SM_	0.908 (0.866, 0.937)	<0.001	0.944 (0.918, 0.962)	<0.001
D_HM_	0.923 (0.888, 0.948)	<0.001	0.952 (0.928, 0.967)	<0.001

ICC, interclass correlation coefficient; CI, confidence interval.

### 3.2. Age-related changes in morphological features of MC

**Figure 3:**
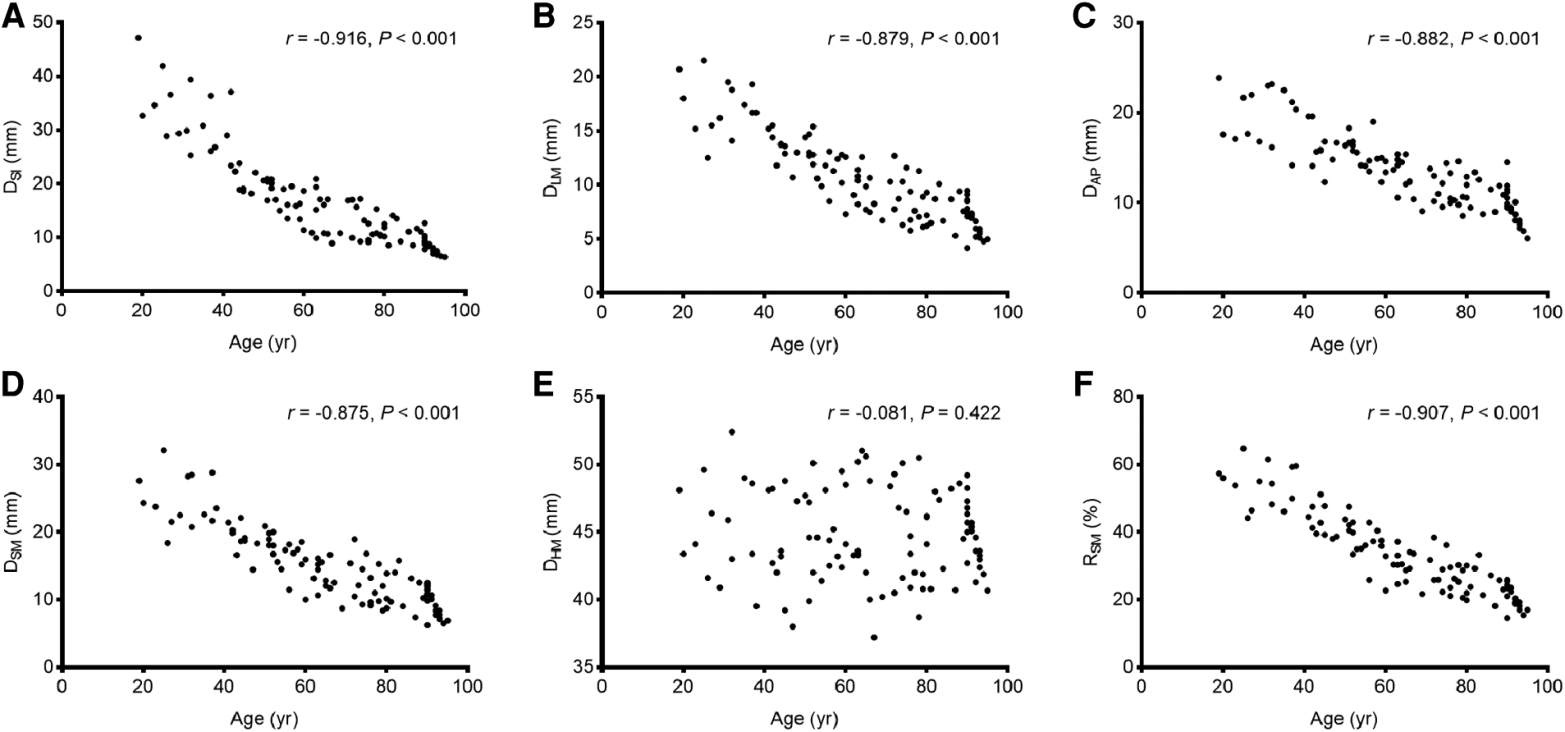
The correlations between age and the values of the parameters of the morphological features. **(A)** D_SI_; **(B)** D_LM_; **(C)** D_AP_; **(D)** D_SM_; **(E)** D_HM_; **(F)** R_SM_.

**Figure 4:**
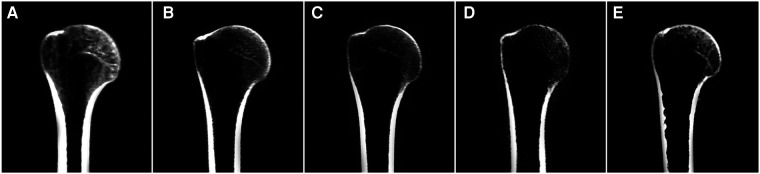
Difference in morphological features of medial column for representative individuals of five different age groups in frontal reconstruction view. **(A)** Group I; **(B)** Group II; **(C)** Group III; **(D)** Group IV; **(E)** Group V.

**Figure 5:**
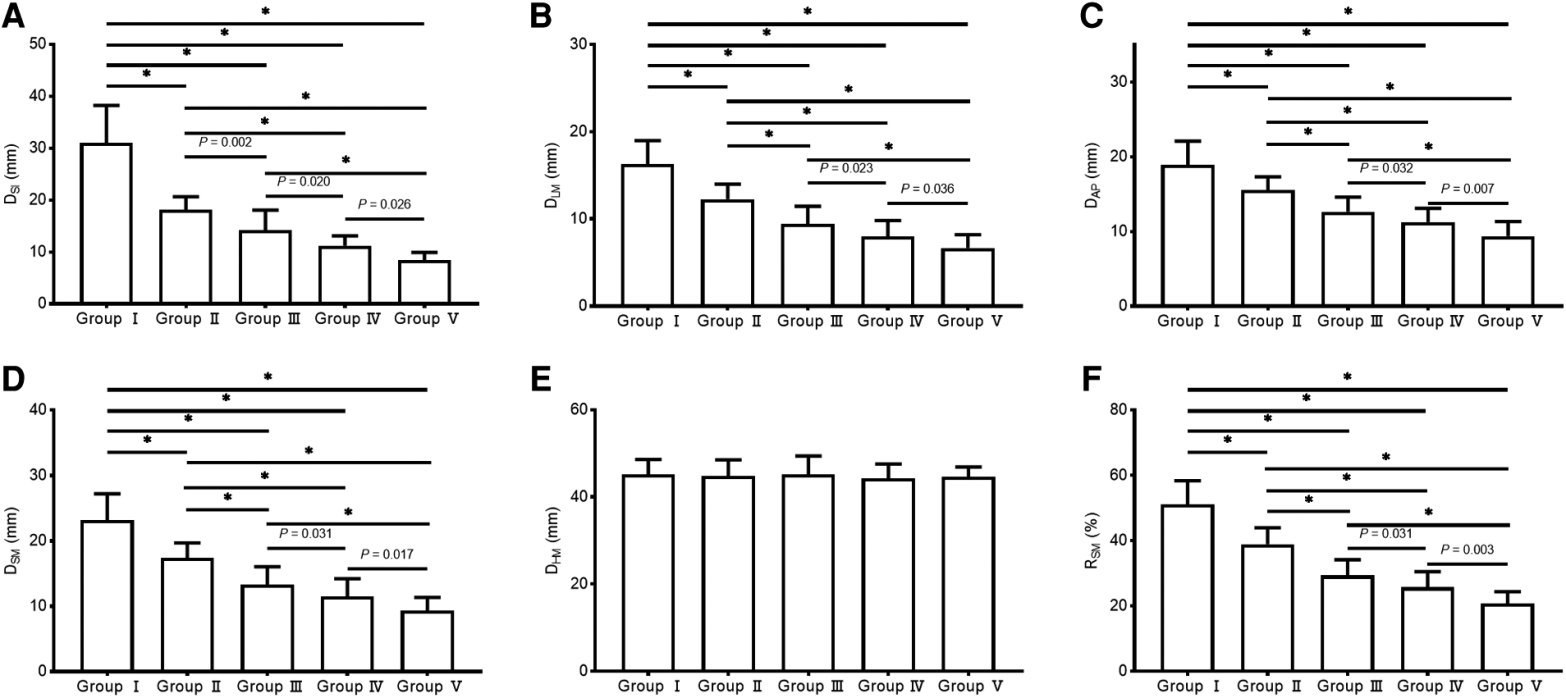
The values of the parameters of the morphological features of the medial column in different age groups. **(A)** D_SI_; **(B)** D_LM_; **(C)** D_AP_; **(D)** D_SM_; **(E)** D_HM_; **(F)** R_SM_.

Significant negative correlations were observed between age and the value of most parameters (including D_SI_, D_LM_, D_AP_, D_SM_, and R_SM_ [all *P* < 0.001], but not D_HM_ [*P* = 0.422]). The parameter values of the morphological features for the study individuals, and correlations between age and parameters, are provided in Table 2 and Figure 3. Difference in morphological features of medial column in five age groups is showed in Figure 4. Comparisons among multiple groups revealed differences in the parameter values of the morphological features of MC among the groups (including D_SI_, D_LM_, D_AP_, D_SM_, and R_SM_, all *P* < 0.001; Table 2). No significant difference was observed in D_HM_ among the groups (*P* = 0.921; Table 2). Pairwise comparisons revealed that the highest mean parameter values of the morphological features were observed in the youngest group (18–44 years), while the oldest group (≥90 years) had the lowest mean parameter values (including D_SI_, D_LM_, D_AP_, D_SM_, and R_SM_, all *P* < 0.001 [Group I vs. Group V]). The values and differences in the parameters of the morphological features among the age groups are shown in Table 3 and Figure 5.

**Table 2:** Parameter values of the morphological feature of the medial column.

Parameters	Median (P_25_, P_75_)	Min	Max
D_SI_ (mm)	14.55 (9.90, 20.33)	6.35	47.20
D_LM_ (mm)	9.99 (7.33, 12.98)	4.17	21.50
D_AP_ (mm)	13.40 (10.33, 15.88)	6.06	23.90
D_SM_ (mm)	14.45 (10.30, 18.63)	6.22	32.09
D_HM_ (mm)	44.45 (42.08, 48.08)	37.20	52.40
R_SM_ (%)	30.34 (23.45, 41.04)	14.57	64.70

P_25_, 25% quartile; P_75_, 75% quartile; Min, the minimal value; Max, the maximal value.

**Table 3:** Parameter values of the morphological features of MC and age in different age groups.

	D_SI_ (mm)	D_LM_ (mm)	D_AP_ (mm)	D_SM_ (mm)	D_HM_ (mm)	R_SM_ (%)	Age (yr)
Group I	31.03 ± 7.19 (27.66–34.39)	16.33 ± 2.68 (15.07–17.58)	18.91 ± 3.17 (17.43–20.39)	23.16 ± 4.03 (21.28–25.04)	45.19 ± 3.44 (43.58–46.79)	51.17 ± 7.22 (47.79–54.55)	33.35 ± 8.15 (29.53–37.17)
Group II	18.13 ± 2.51 (16.96–19.30)	12.28 ± 1.71 (11.48–13.09)	15.55 ± 1.76 (14.72–16.37)	17.42 ± 2.25 (16.37–18.47)	44.81 ± 3.70 (43.08–46.54)	38.99 ± 5.00 (36.64–41.33)	52.55 ± 4.26 (50.56–54.54)
Group III	14.20 ± 3.86 (12.39–16.00)	9.46 ± 1.99 (8.53–10.39)	12.63 ± 1.97 (11.71–13.55)	13.31 ± 2.75 (12.03–14.60)	45.15 ± 4.24 (43.16–47.13)	29.38 ± 4.79 (27.13–31.62)	66.60 ± 4.63 (64.43–68.77)
Group IV	11.22 ± 1.87 (10.35–12.10)	8.01 ± 1.80 (7.16–8.85)	11.26 ± 1.82 (10.41–12.11)	11.49 ± 2.70 (10.23–12.76)	44.32 ± 3.27 (42.78–45.85)	25.75 ± 4.78 (23.52–27.99)	80.70 ± 4.24 (78.71–82.69)
Group IV	8.39 ± 1.54 (7.67–9.11)	6.67 ± 1.53 (5.96–7.39)	9.35 ± 1.98 (8.43–10.28)	9.32 ± 2.03 (8.37–10.27)	44.59 ± 2.31 (43.51–45.67)	20.76 ± 3.61 (19.07–22.45)	91.50 ± 1.57 (90.76–92.24)
*P* value*	<0.001	<0.001	<0.001	<0.001	0.921	<0.001	<0.001

* Comparison among multiple groups was performed with one-way analysis of variance. Data are reported as mean ± standard deviation (95% confidence interval).

### 3.3. Morphological grading of MC

The highest mean value of R_SM_ was observed in the baseline group (R_SM_ = 51.17 ± 7.22% in Group I), and it was set as the reference value for morphological grading. Between-grades comparisons were performed to select the effective thresholds, which could distinguish between the different morphological grades. The threshold values of grading were set at different times of SD below the mean value of R_SM_ in the baseline group (from 0.5SD to 4SD with 0.5SD interval). Finally, 1.5SD and 3SD below the mean value of R_SM_ in Group I (R_SM_ = 40.34%, R_SM_ = 29.51%) were selected as the thresholds for the morphological grading of MC. The specific morphological grading was illustrated as follows: (1) Grade I: R_SM_ > Mean - 1.5SD (R_SM_ >40.34%); (2) Grade II: Mean - 1.5SD ≤ R_SM_ ≤ Mean - 3SD (29.51% ≤ R_SM_ ≤ 40.34%); and (3) Grade III: R_SM_ < Mean - 3SD (R_SM_ <29.51%). There were significant differences in the value of morphological features parameters of MC among the three grades (including D_SI_, D_LM_, D_AP_, D_SM_, and R_SM_, all *P* < 0.05; Table 4), while no significant difference was observed regarding sex (*P* = 0.329; Table 4).

**Table 4:** Baseline characteristics and morphological features of the medial column among the grades.

	Grade I (*n* = 19)	Grade II (*n* = 35)	Grade III (*n* = 46)	*P* value
Age (yr)	33.37 ± 8.91 (29.07–37.66)	59.17 ± 10.99 (55.40–62.95)^§^	82.37 ± 10.49 (79.26–85.48)^§‖^	<0.001*
Sex (Female/male)	11/8	14/21	25/21	0.329†
D_SI_ (mm)	29.40 (25.30, 36.40)	17.10 (15.20, 20.40)	9.82 (8.52, 10.88)^§‖^	<0.001^‡^
D_LM_ (mm)	16.20 (14.10, 18.80)	11.80 (10.40, 13.00)	7.19 (6.08, 8.47)^§‖^	<0.001^‡^
D_AP_ (mm)	17.70 (16.20, 22.00)	14.80 (13.80, 16.40)	10.15 (9.04, 11.60)^§‖^	<0.001^‡^
D_SM_ (mm)	22.50 (20.74, 27.60)	16.64 (15.22, 18.31)	10.11 (8.63, 11.77)^§‖^	<0.001^‡^
D_HM_ (mm)	44.68 ± 3.81 (42.85–46.52)	45.87 ± 3.53 (44.66–47.08)	44.05 ± 2.95 (43.18–44.93)	0.071*
R_SM_ (%)	51.16 (47.49, 57.38)	36.15 (33.12, 39.92)	23.39 (20.22, 25.84)^§‖^	<0.001^‡^

* Comparison among grades was performed with one-way analysis of variance.

^†^ Comparison among grades was performed with *χ*^2^ test.

^‡^ Comparison among grades was performed with Kruskal–Wallis *H* test.

^§^ Compared to Grade I, *P* < 0.001 (LSD post-hoc test or Kruskal–Wallis H test [*P* values were adjusted with Bonferroni correction]).

^||^ Compared to Grade II, *P* < 0.001 (LSD post-hoc test or Kruskal–Wallis H test [*P* values were adjusted with Bonferroni correction]).

Significant differences in the parameters of the morphological features were detected between Grade I and Grade II (D_SI_, *P* = 0.010; D_LM_, *P* = 0.013; D_AP_, *P* = 0.024; D_SM_, *P* = 0.006; R_SM_, *P* = 0.003; All *P* values were adjusted with Bonferroni correction). Age and D_HM_ are presented as mean ± standard deviation (95% confidence interval); the other parameters of morphological features (including D_SI_, D_LM_, D_AP_, D_SM_, and R_SM_) are presented as median (25% quartile, 75% quartile).

## 4. Discussion

This study investigated the age-related changes in the morphological features of MC and presented morphological grading based on an objective definition of MC that covers a broader age range. We identified and quantified the age-related changes in the morphological features of MC with an explicit definition. The parameter values of the morphological features decreased in multiple dimensions with increasing age. The highest mean values of the parameters of the morphological features (D_SI_ = 31.03 mm, D_LM_ = 16.33 mm, D_AP_ = 18.91 mm, D_SM_ = 23.16 mm, R_SM_ = 51.17%) were observed in young adults (18–44 years) and decreased incrementally with increasing age until the lowest mean values (D_SI_ = 8.39 mm, D_LM_ = 6.67 mm, D_AP_ = 9.35 mm, D_SM_ = 9.32 mm, R_SM_ = 20.76%) were observed in individuals with advanced age (≥90 years). Additionally, a morphological grading of MC was presented based on the thresholds with 1.5 SD and 3 SD below the mean value of R_SM_ of young adults (R_SM_ = 40.34%, R_SM_ = 29.51%; Group I: 18–44 years).

The importance of MC for PHF management is widely acknowledged; however, there is no consensus regarding the definition of MC. Sprecher et al. (4) and Wang et al. (18) defined MC as the trabecular or cortical region using the humeral head height as a reference. Helfen et al. (5) chose a certain range of high-resolution peripheral quantitative CT scans (150 sections). Russo et al. (17) selected 20–25 mm long medial metaphysis without elucidating the anatomical rationale. However, the definitions of MC in the aforementioned studies varied by using certain distances defined subjectively as the references rather than the morphological features of MC. Additionally, the cortical or trabecular bone alone may not fully account for age-related changes in MC, as MC is affected by cortical and trabecular bone loss, which may be an early sign of a decrease in the mechanical properties of MC (4, 5, 18, 27). Therefore, using the endocortical longitudinal trabecular region as a reference, we defined MC as the osseous region in the medial metaphysis combining the endocortical trabecular bone and adjacent cortical bone, that is dynamic with age. This definition, which is explicit with a reasonable anatomical rationale, could comprehensively reflect the age-related changes in MC.

In the present study, all values of the parameter of the morphological features of MC decreased with increasing age. Compared with younger individuals (18–44 years), older individuals (≥60 years) exhibited more pronounced decrease in the parameter values of the morphological features, especially in the advanced age population (≥90 years). Our findings specifically quantify the age-related changes in MC regarding the morphological features and support a previous study that showed a considerable loss of cortical and trabecular bone of the metaphysis with increasing age (5). This finding was also indirectly supported by a previous histomorphometric study, which showed that the bone density of the trabecular bone in the medial metaphysis decreased significantly in osteoporotic individuals. Additionally, we observed that parameter value decrease ‘of the morphological features tended to flatten after the age of 75 years. Similarly, Helfen et al. (5) showed that decreases in the values of the microstructural parameters of the metaphysis were not visible after the age of 80 years.

Morphological grading based on morphological feature changes could provide important references for fracture management. The Singh index—which describes the morphological changes in the trabecular bone of the femoral neck and head—has an important influence on risk stratification and prognosis prediction of fracture (21, 22, 33). Previous studies have reported a lower Singh index common in patients with hip or subsequent contralateral fractures. Carow et al. (33) demonstrated that a Singh index ≤ 3 was a risk factor for in-hospital mortality (OR = 5.00). In this study, we presented morphological grading of MC which was modeled after the grading of bone mineral density and vertebral compressive fractures (29, 30), using the mean value of R_SM_ of young adults (Group I: 18–44 years) as the reference. MC was categorized into three grades: < 1.5 SD (R_SM_ > 40.34%), 1.5 SD – 3.0 SD (29.51% ≤ R_SM_ ≤ 40.34%), and > 3 SD (R_SM_ < 29.51%) below the mean value of R_SM_ of young adults. Between-grades comparison verified the validation of the morphological grading preliminarily.

Our study has some limitations; first, the morphological grading presented in our study was based on the Chinese population with a limited sample size; thus, further validation is required to extend the validity of its results to other races. Second, the measurements of morphological parameters were obtained from the CT images acquired with only one CT scanner and scanning protocol. The clinical application of our method of measuring parameters needs to be verified using CT images acquired with different scanning protocols. However, this did not hinder the feasibility of the measurement method and comprehensive insight into MC in this study. Lastly, additional biomechanical and clinical studies are needed for further validation; however, this is beyond the scope of this study.

## 5. Conclusion

This study presents the age-related changes in the morphological features of MC of PH and morphological grading, based on an explicit definition of MC. Our results show that the parameter values of the morphological features of MC decreased with increasing age. Thus, the morphological features of MC could be categorized into three grades. This study may provide a more comprehensive insight into age-related changes in the morphological features of MC that can facilitate risk stratification and optimize the management of PHFs.

## Data Availability

The raw data supporting the conclusions of this article will be made available by the authors, without undue reservation.
